# A hybrid in silico/in-cell controller for microbial bioprocesses with process-model mismatch

**DOI:** 10.1038/s41598-023-40469-y

**Published:** 2023-09-04

**Authors:** Tomoki Ohkubo, Yuki Soma, Yuichi Sakumura, Taizo Hanai, Katsuyuki Kunida

**Affiliations:** 1 Graduate School of Science and Technology, Nara Institute of Science and Technology, Ikoma, Nara 8916-5 Japan; 2 Laboratory for Synthetic Biology, Graduate School of Bioresource and Bioenvironmental Sciences, Kyushu University, W5-729, 744, Motooka, Nishi-ku, Fukuoka, 819-0395 Japan; 3 Data Science Center, Nara Institute of Science and Technology, Ikoma, Nara 8916-5 Japan; 4 School of Medicine, Fujita Health University, Toyoake, Aichi 470-1192 Japan

**Keywords:** Systems biology, Computer modelling, Control theory, Synthetic biology

## Abstract

Bioprocess optimization using mathematical models is prevalent, yet the discrepancy between model predictions and actual processes, known as process-model mismatch (PMM), remains a significant challenge. This study proposes a novel hybrid control system called the hybrid in silico/in-cell controller (HISICC) to address PMM by combining model-based optimization (in silico feedforward controller) with feedback controllers utilizing synthetic genetic circuits integrated into cells (in-cell feedback controller). We demonstrated the efficacy of HISICC using two engineered *Escherichia coli* strains, TA1415 and TA2445, previously developed for isopropanol (IPA) production. TA1415 contains a metabolic toggle switch (MTS) to manage the competition between cell growth and IPA production for intracellular acetyl-CoA by responding to external input of isopropyl β-d-1-thiogalactopyranoside (IPTG). TA2445, in addition to the MTS, has a genetic circuit that detects cell density to autonomously activate MTS. The combination of TA2445 with an in silico controller exemplifies HISICC implementation. We constructed mathematical models to optimize IPTG input values for both strains based on the two-compartment model and validated these models using experimental data of the IPA production process. Using these models, we evaluated the robustness of HISICC against PMM by comparing IPA yields with two strains in simulations assuming various magnitudes of PMM in cell growth rates. The results indicate that the in-cell feedback controller in TA2445 effectively compensates for PMM by modifying MTS activation timing. In conclusion, the HISICC system presents a promising solution to the PMM problem in bioprocess engineering, paving the way for more efficient and reliable optimization of microbial bioprocesses.

## Introduction

A primary goal of bioprocess engineering is to produce a larger amount of a desired product at higher rates from a smaller amount of raw materials^1,2^. To achieve this, it is necessary to control the behavior of microorganisms so that their capabilities can be harnessed to the fullest extent. Approaches to this can be broadly classified into two categories: computerized process control (in silico feedforward control) and autonomous feedback control by synthetic genetic circuits integrated in cells (in-cell feedback control)^3–5^.

In silico feedforward control is an approach widely used in industries where mathematical models are used to predetermine the optimal values of inputs to the process, such as temperature, pH, and substrate and inducer feeds^6–10^. In silico feedforward control comprises a sophisticated approach that maximizes product yield by predicting the future process state and managing the various tradeoffs that arise with respect to process inputs. However, one challenge is that the predetermined input values are no longer optimal in the actual process when there is a significant mismatch between the model and the actual process (process-model mismatch, PMM). One solution to the PMM problem is model predictive control (MPC), in which the state variables of the ongoing process are measured in real-time and fed back to the control inputs^11–19^. Although MPC is widely used for process control, it can be unavailable if the model includes the intracellular concentrations of RNA, metabolites, or enzymes as state variables that are difficult to monitor online.

In-cell feedback control is a nascent approach that emerged from synthetic biology^20^. In the last two decades, various examples of synthetic genetic circuits have been reported, such as those designed to control cell density^21–24^, co-culture composition^25,26^, or intracellular protein expression levels^27–31^. Unlike in silico controllers, in-cell controllers can only provide simple feedback control, such as proportional control; sophisticated control to maximize future product yields is difficult for in-cell controllers. Conversely, it can detect intracellular RNAs, enzymes, and metabolites, which are difficult to monitor using process sensors or biochemical analyses and provide feedback on cell behavior in situ. Therefore, the in silico model-based controller and the in-cell feedback controller complement each other's limitations.

**Figure 1:**
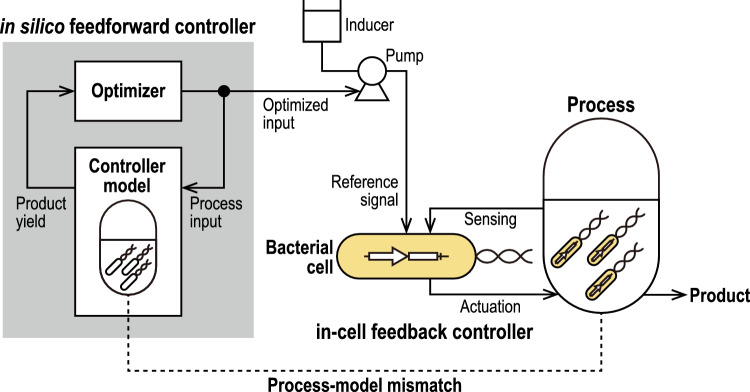
Conceptual diagram of the hybrid in silico/in-cell controller (HISICC). The in silico feedforward controller calculates the optimal control inputs for the process based on the controller model. An example of a control input is the inducer feed. The bacterial cells receive this control input as a reference signal and autonomously perform feedback control.

To overcome the PMM problem of in silico controllers, we propose a hybrid control strategy that combines a high-level in silico feedforward controller and a low-level in-cell controller (hybrid in silico/in-cell controller, HISICC) (Fig. 1). When the actual process state deviates from the prediction by the in silico controller owing to the PMM, the in-cell feedback controller senses the actual state and corrects the cell behavior to prevent a decrease in the product yield based on the sensing. To demonstrate the concept of HISICC, this study focuses on the isopropanol (IPA) production process using the two engineered *Escherichia coli* strains we reported on previously^32,33^ as an example of the bioprocess. As described in detail in the Results section, prediction error in cell growth is a critical PMM in this process, which leads to decrease in IPA yield. Since only one of these strains contains an in-cell feedback controller which detects cell density, this strain can be defined as an example of HISICC in combination with the in silico feedforward controller whereas the other strain cannot. The study goals were (1) to construct a new mathematical model for an in silico feedforward controller and assemble an HISICC by coupling it with a previously developed in-cell feedback controller and (2) to demonstrate the robustness of the HISICC to PMM, namely prediction errors in cell growth, by comparing the two strains in terms of IPA yield in multi-round simulations where various magnitudes of PMM were assumed.

## Results

### IPA production process using two engineered strains

Prior to describing the details of mathematical modeling for the design of in silico feedforward controllers, we provide an overview of the IPA production process using the two engineered strains that we previously developed, TA1415 and TA2445^32,33^. In conventional IPA production processes, cell growth and IPA production compete for intracellular acetyl-CoA synthesized from the substrates. This competition needs to be balanced since an imbalance in the use of intracellular acetyl-CoA for either cell growth or IPA production results in reduced IPA yield. TA1415 has a genetic circuit called the metabolic toggle switch (MTS) that allows this competition to be managed by an external input of an inducer, isopropyl β-d-1-thiogalactopyranoside (IPTG). We designed an in silico feedforward controller that optimizes the IPTG input using a mathematical model of the strain. However, because TA1415 does not have an in-cell feedback controller, the combination of TA1415 and the in silico controller does not comprise an HISICC. In contrast, TA2445 has an in-cell feedback controller consisting of an MTS and another genetic circuit to detect cell density, termed quorum sensing. Owing to the in-cell feedback controller, TA2445 autonomously controls cell growth and IPA production in accordance with the external IPTG input as a reference signal. Therefore, the combination of TA2445 and an in silico feedforward controller designed for this strain can be considered an example of HISICC.

In both strains, the activated MTS stopped the synthesis of citrate synthase (the enzyme that mediates the reaction that initiates the TCA cycle) and simultaneously initiates the synthesis of a series of enzymes for IPA production, thereby achieving a changeover from cell growth to IPA production. The timing of MTS activation creates a tradeoff: if the MTS is activated too early, the IPA yield is low because the cells do not grow sufficiently; if the MTS is activated too late, the IPA yield is also low because extracellular nutrients are used up by cell growth, resulting in insufficient synthesis of a series of enzymes for IPA production.

**Figure 2:**
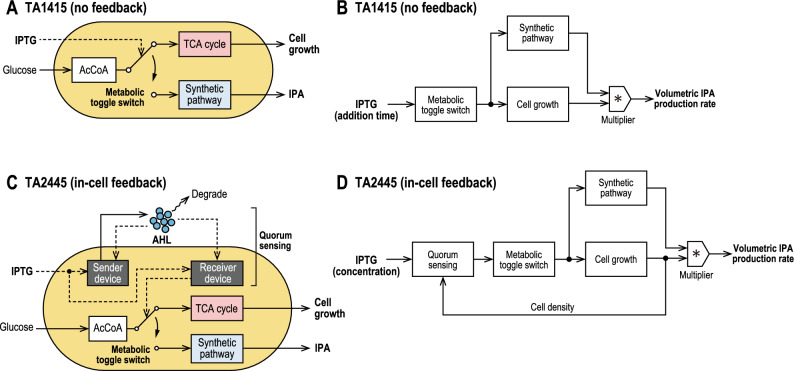
Genetic circuits of TA1415 and TA2445. (**A**) Genetic circuit of TA1415, in which the metabolic toggle switch (MTS) changes the flow of intracellular Acetyl-CoA (AcCoA) from the TCA cycle to the synthetic pathway for isopropanol (IPA) production; the MTS is activated when IPTG is added to the medium. (**B**) Block diagram showing the control structure of TA1415, in which the MTS changes the expression level of the synthetic pathway and cell growth. The volumetric production rate of IPA is proportional to the product of cell density and the expression level of the synthetic pathway. (**C**) Genetic circuit of TA2445 with the sender device to secrete and the receiver device to detect AHL, which realize quorum sensing collectively; the MTS is activated when the receiver device detects an increased extracellular concentration of AHL due to cell growth. (**D**) Block diagram showing the control structure of TA2445. Quorum sensing provides feedback of increased cell density to the MTS, the sensitivity of which depends on IPTG concentration in the medium.

In TA1415 cells, the MTS was activated by the addition of IPTG to the medium in the middle of the culture period (Fig. 2A). Therefore, the timing of IPTG addition can be defined as the input variable of the process to be optimized (Fig. 2B).

TA2445 has an additional genetic circuit for quorum sensing that detects cell density to activate the MTS, as described in the Introduction (Fig. 2C). The circuit is composed of an intercellular messenger called an acylated homoserine lactone (AHL) and genetic devices that send or receive it. As the cell density increases, so does the AHL concentration in the medium. When the AHL concentration reaches a certain level, the receiver device detects AHL and activates the MTS. The sender and receiver devices utilize the same promoter, which responds to both IPTG and AHL. This allows the sensitivity of quorum sensing to be tuned by varying the extracellular concentration of IPTG. Thus, IPTG concentration can be defined as the input variable of the process to be optimized (Fig. 2D); if IPTG concentration is too high, quorum sensing becomes too sensitive, and the MTS is activated too early. Conversely, if the IPTG concentration is too low, quorum sensing becomes too insensitive, and the MTS is activated too late or is not activated at all.

### Mathematical modeling

#### TA1415 model

The TA1415 model is based on a two-compartment model, which is a type of structured model constructed by Williams that divides the cells into two compartments: XA and XG^34^. The XA compartment represents the active part of cells directly involved in cell growth, including RNA, ribosomes, and small metabolites such as amino acids. On the other hand, the XG compartment represents an inactive part that is not directly involved in cell growth, including DNA, proteins, and cell membranes. XA is produced from the extracellular substrate S, and XG is produced from XA. Since the amount of XG per cell is nearly constant, it can be considered proportional to the cell density. Williams’ two-compartment model, although quite simple, can explain the lag phase as well as the experimental fact that cell growth continues for a period after removal of the substrate from the medium in the middle of the log phase. In the simulation, no additional XA is produced after substrate removal, while XG is produced until the XA present in the cells is exhausted.

**Figure 3:**
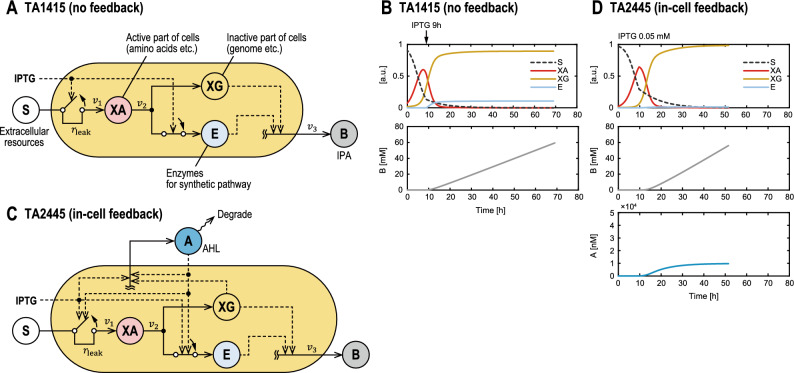
The three-compartment models of the two strains. (**A**) TA1415 model. Active compartment XA is synthesized by the TCA cycle from S, which represents extracellular resources; from XA, inactive compartment XG and E, a series of enzymes on the synthetic pathway for IPA production, are synthesized. When activated, the MTS stops synthesizing XA and initiates synthesis of E. The production rate of IPA, represented by B is proportional to XG and E. (**B**) Dynamics of state variables for TA1415 model, with IPTG added at 9 h to activate the MTS. (**C**) TA2445 model. A, which represents extracellular AHL, increases with cell growth. The MTS is activated when A reaches a certain level that depends on extracellular IPTG concentration. (**D**) Dynamics of state variables for TA2445 model at IPTG concentration of 0.05 mM.

When IPTG is fed to TA1415 cells, cell growth slows but does not immediately stop^32^. This behavior is similar to that observed after substrate removal during the log phase, as described above. This suggests that when the MTS stops the TCA cycle, cells store materials for cell growth, such as amino acids, which can be used to continue cell growth. Thus, to model the MTS, we extended Williams' two-compartment model to a three-compartment model with an additional compartment, E, representing a series of enzymes for IPA production (Fig. 3A).

Hereinafter, we describe the model equations. The initial values of the state variables and parameters are summarized in Tables 1 and 2, respectively. The mass balance equations are as follows:

1 $$\begin{array}{c}\frac{dS}{dt}={-v}_{1}\end{array}$$

2 $$\begin{array}{c}\frac{d{X}_{A}}{dt}={v}_{1}-{v}_{2}\end{array}$$

3 $$\begin{array}{c}\frac{d{X}_{G}}{dt}={v}_{2}\left(1-{a}_{E}u\right)\end{array}$$

4 $$\begin{array}{c}\frac{dE}{dt}={v}_{2}{a}_{E}u\end{array}$$

5 $$\begin{array}{c}\frac{dB}{dt}={v}_{3}\end{array}$$

where ${X}_{A}$, ${X}_{G}$ and $E$ represent the three aforementioned compartments, XA, XG, and E, respectively. $B$ represents the IPA concentration in the medium. $S$, unlike in Williams’ model, represents collective extracellular resources, including not only glucose but also waste accumulation and pH shifts, as discussed in the “Discussion” section. $u$ indicates the IPTG concentration, that is, the control input. $u$ is normalized and takes either 0 or 1. By incorporating $u$ into the mass balance and reaction rate equations described below, the changeover of the reactions due to the MTS is mathematically expressed. ${a}_{E}$ represents the allocation of XA for the synthesis of XG and E, as described below.

**Table 1:** State and input variables for the two models.

Symbol	Initial value for TA1415	Initial value for TA2445	Unit	Description
For TA1415 and TA2445				
$$ S$$	$$1-{X}_{A}(0)-{X}_{G}(0)$$	$$1-{X}_{A}(0)-{X}_{G}(0)$$	Dimensionless	Extracellular resources
${ X}_{A}$	Estimated	Estimated	Dimensionless	Active compartment
${ X}_{G}$	0.02	0.005	Dimensionless	Inactive compartment
$$ B$$	0	0	mM	IPA concentration
$$ E$$	0	0	Dimensionless	Enzymes for IPA production
For TA1415				
$$ u$$	–	–	Dimensionless	Normalized IPTG concentration
For TA2445				
$$ A$$	–	0.01	nM	AHL concentration
$$ u$$	–	–	mM	IPTG concentration

**Table 2:** Parameters for the two models estimated with all datasets.

Symbol	Value for TA1415	Value for TA2445	Unit	Description
For TA1415 and TA2445				
${ k}_{1}$	0.4772	0.4526	/h	Production rate parameter for Reaction 1
${ k}_{2}$	0.9089	1	/h	Production rate parameter for Reaction 2
${ k}_{3}$	1.2418	2	mM/h	Production rate parameter for Reaction 3
${ r}_{leak}$	0.2777	0.0607	Dimensionless	Leak ratio of Reaction 1
${ a}_{E}$	0.1508	0.0190	Dimensionless	Ratio of XA allocated to the synthesis of E
${ K}_{E}$	0.0081	0.0034	Dimensionless	Saturation coefficient for E in production rate of B
$$ {X}_{A}(0)$$	0.0572	0.0240	Dimensionless	Initial value of XA. Ten times of $${X}_{G}(0)$$ was set as the upper limit of estimation
${ N}_{m}$	5.4651	5.6799	OD600	Carrying capacity
For TA2445				
${ k}_{A}$	–	1.9137E+3	nM/h	AHL production rate parameter
${ d}_{A}$	–	0.1489	/h	AHL degradation rate parameter
${ K}_{A}$	–	1.596	nM	Dissociation constant for AHL (estimated with TA2946 data)
${ K}_{u}$	–	0.02268	mM	Dissociation constant for IPTG (estimated with TA2946 data)
${ n}_{A}$	–	1.752	Dimensionless	Hill coefficient for AHL (estimated with TA2946 data)
${ n}_{u}$	–	1.597	Dimensionless	Hill coefficient for IPTG (estimated with TA2946 data)

${v}_{1}$, ${v}_{2}$, and ${v}_{3}$ represent the rates of three reactions (Reactions 1–3), respectively. First, in Reaction 1, S is consumed, and XA is synthesized by the TCA cycle:

6 $$\begin{array}{c}{v}_{1}={k}_{1}S\left({X}_{A}+{X}_{G}+E\right)\left(1-\left(1-{r}_{\mathrm{leak}}\right)u\right)\end{array}$$

The reaction rate ${v}_{1}$ is proportional to both the total cell size ${X}_{A}+{X}_{G}+E$ and $S$. ${r}_{\mathrm{leak}}$ is a parameter that describes the incomplete MTS changeover; it allows Reaction 1 to proceed slowly and XA to be synthesized even in the presence of IPTG. In Reaction 2, XA is consumed and XG and E are synthesized as follows:

7 $$\begin{array}{c}{v}_{2}={k}_{2}{X}_{A}\left({X}_{G}+E\right)\end{array}$$

The ratios of the XG and E production rates are represented by ${a}_{E}$ in Eqs. (3) and (4). The reaction rate ${v}_{2}$ is proportional to ${X}_{A}$ and the remainder of the cell, ${X}_{G}+E$. ${k}_{1}$ and ${k}_{2}$ are both reaction rate parameters that govern the rate of XG synthesis, that is, the cell growth rate, through Reactions 1 and 2 in tandem. In Reaction 3, IPA (represented by $B$) is synthesized, and its reaction rate ${v}_{3}$ depends on ${X}_{G}$ and $E$:

8 $$\begin{array}{c}{v}_{3}={k}_{3}\frac{E}{E+{K}_{E}}{X}_{G}\end{array}$$

We introduced a saturation constant ${K}_{E}$ so that the rate of Reaction 3 was saturated with respect to E. Finally, the observation equations that link the state variables of the model to the measurements of cell density (OD600) and IPA concentration are as follows:

9 $$\begin{array}{c}{y}_{1}={N}_{m}{X}_{G}\end{array}$$

10 $$\begin{array}{c}{y}_{2}=B\end{array}$$

where ${y}_{1}$, ${y}_{2}$, and ${N}_{m}$ represent the cell density, IPA concentration, and constant proportionality between the cell density and XG, respectively. Simulated trajectories of each compartment at IPTG concentration of 0.05 mM are shown in Fig. 3B. When MTS is not activated ($u=0$), Reaction 1 proceeds, and only XG is produced in Reaction 2. When MTS is activated ($u=1$), Reaction 1 stops, and E is produced in addition to XG in Reaction 2 (Fig. 3B). However, because of some leakage in the promoter, represented by ${r}_{\mathrm{leak}}$, Reaction 1 does not stop completely, and a small amount of XA continues to be produced thereafter.

#### TA2445 model

The TA2445 model combines the three-compartment TA1415 model with a portion of the quorum sensing model constructed by You et al. (Fig. 3C)^23^. The mass balance equations are as follows:

11 $$\begin{array}{c}\frac{dS}{dt}={-v}_{1}\end{array}$$

12 $$\begin{array}{c}\frac{d{X}_{A}}{dt}={v}_{1}-{v}_{2}\end{array}$$

13 $$\begin{array}{c}\frac{d{X}_{G}}{dt}={v}_{2}\left(1-{a}_{E}z\right)\end{array}$$

14 $$\begin{array}{c}\frac{dE}{dt}={v}_{2}{a}_{E}z\end{array}$$

15 $$\begin{array}{c}\frac{dB}{dt}={v}_{3}\end{array}$$

16 $$\begin{array}{c}\frac{dA}{dt}={k}_{A}{X}_{G}z-{d}_{A}A\end{array}$$

Equations (11–15) are nearly identical to the mass balance equations for the TA1415 (Eqs. 1–5), although the IPTG input $u$ is replaced by the promoter output $z$ of the receiver device in the in-cell feedback circuit. This represents the activation of the MTS by the in-cell feedback circuit; as the concentration of AHL increases, the MTS is activated, and its threshold is regulated via the IPTG concentration. Thus, $z$ is a two-variable function of AHL and IPTG concentrations, expressed as follows: Eq. (16) is the mass balance equation for the AHL, represented by $A$, adopted from the model of You et al. The first term representing AHL production includes $z$ because the same promoter is used in the sender and receiver devices. Therefore, positive feedback occurs during AHL production. The second term represents the decomposition of AHL (first-order reaction), where ${d}_{A}$ is the decomposition rate parameter of AHL. The reaction rate and observation equations are identical to those of the TA1415 model, except that $u$ is replaced by $z$.

17 $$\begin{array}{c}{v}_{1}={k}_{1}S\left({X}_{A}+{X}_{G}+E\right)\left(1-\left(1-{r}_{\mathrm{leak}}\right)z\right)\end{array}$$

18 $$\begin{array}{c}{v}_{2}={k}_{2}{X}_{A}\left({X}_{G}+E\right)\end{array}$$

19 $$\begin{array}{c}{v}_{3}={k}_{3}\frac{E}{E+{K}_{E}}{X}_{G}\end{array}$$

20 $$\begin{array}{c}{y}_{1}={N}_{m}{X}_{G}\end{array}$$

21 $$\begin{array}{c}{y}_{2}=B\end{array}$$

**Figure 4:**
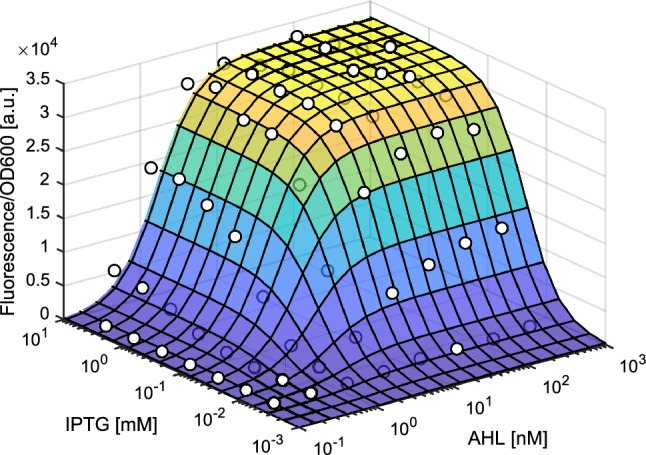
Response to IPTG and AHL of the promoters used in the sender and receiver devices of TA2445. Experimental data (white dots) of the *Escherichia coli* strain TA2946, which has a plasmid with the GFP gene downstream of the same promoter, was fitted using Hill equation.

The promoter response $z$ to IPTG and AHL concentrations was modeled using data from another *E. coli* strain we previously developed, TA2946^33^. TA2946 contains a GFP gene on a plasmid downstream of the same promoter as the sender and receiver devices of TA2445. Using TA2946, we measured the promoter response to IPTG and AHL in terms of fluorescence intensity. The response curve of the promoter was fitted to the Hill equation to obtain the values of its four parameters: dissociation constants and Hill coefficients for IPTG or AHL (Fig. 4).

22 $$\begin{array}{c}z=\frac{{A}^{{n}_{A}}}{{A}^{{n}_{A}}+{{K}_{A}}^{{n}_{A}}}\frac{{u}^{{n}_{u}}}{{u}^{{n}_{u}}+{{K}_{u}}^{{n}_{u}}}\end{array}$$

Approximation of the promoter response using the product of the Hill equations for AHL and IPTG was reported in a previous study using similar promoters^35^. Simulated trajectories of each compartment at IPTG concentration of 0.05 mM are shown in Fig. 3D.

### Model simulation and validation

**Figure 5:**
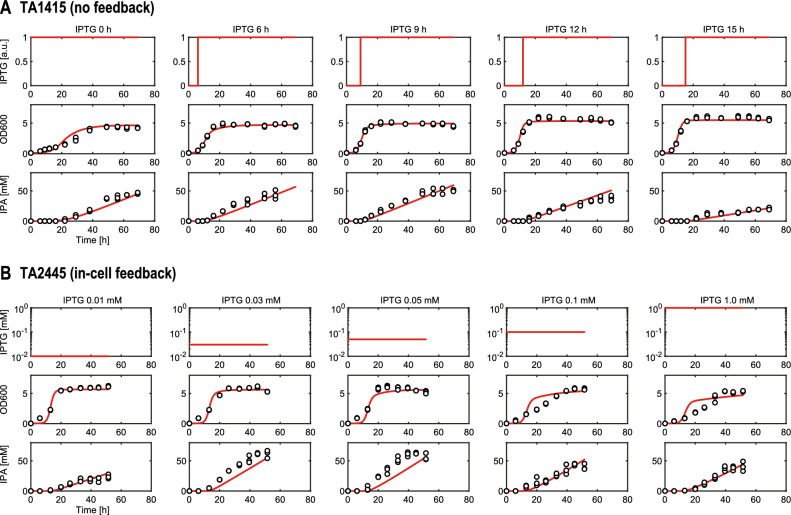
Simulations of the IPA production process for various IPTG inputs using the models of the two strains. Both models were trained using all datasets. White dots represent experimental data and red lines represent simulation results. (**A**) for TA1415. (**B**) for TA2445.

**Figure 6:**
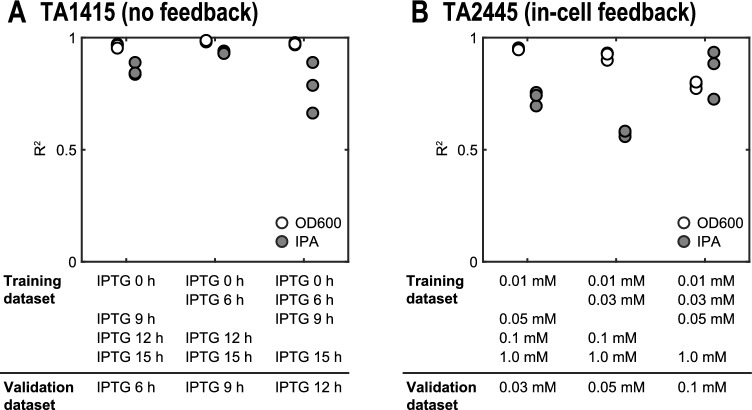
Hold-out validation of the models of the two strains, using a dataset from a flask subjected to one of five IPTG input conditions as a validation dataset. Each circle represents a validation round (18 rounds of validation in total for each strain). Details of the datasets are summarized in Table S2. (**A**) for TA1415. (**B**) for TA2445.

The TA1415 and TA2445 models were trained using experimental data on the IPA production process obtained in previous studies^32,33^. The details of the experimental data are described in the “Materials and methods” section. Both models trained with all experimental datasets fit closely (Fig. 5, Table 2). This indicates that, despite their simple structure, our models capture the dynamics of cell growth and IPA production of the two strains in response to various IPTG inputs. Additionally, we used the hold-out validation method to ensure that the two trained models did not overfit the training data (Fig. 6). The details of the validation method are described in the “Materials and methods” section. The coefficients of determination ${R}^{2}$ were above 0.5 for all test data, indicating that both models have adequate generalization performance within the range of IPTG input values of the training data. The slightly lower ${R}^{2}$ values for IPA concentration than for cell density (OD600) may be because the three-compartment model does not represent the slowdown of IPA production rate due to substrate depletion.

### Model-based input optimization

**Figure 7:**
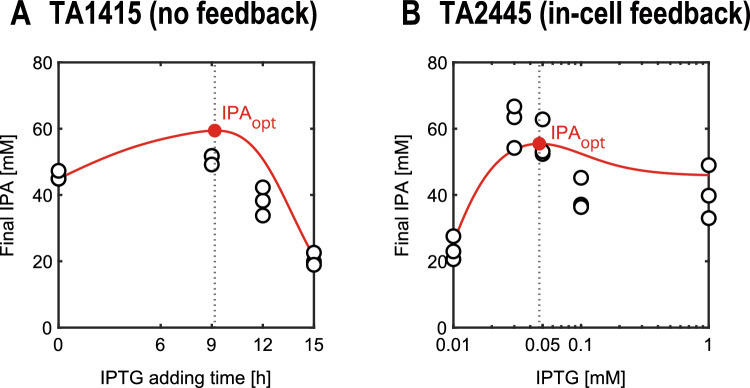
Model-based optimization of IPTG inputs to maximize IPA yield. White and red dots represent experimental data and optimized values, respectively, and red lines represent results of the exhaustive simulations. (**A**) for TA1415. (**B**) for TA2445.

To demonstrate the optimal control by the in silico feedforward controller, we optimized the IPTG input variables to maximize the IPA concentration at the end of the culture using the two models. These models were trained using all datasets before input optimization (Fig. 5, Table 2). The timing of IPTG addition for TA1415 (Fig. 7A) and the concentration of IPTG for TA2445 (Fig. 7B) were optimized. The feasible region for IPTG input was defined as 0–15 h for TA1415 and 0.01–1.0 mM for TA2445. In addition, to visualize the overall distribution of IPA yield over the range of feasible IPTG input values, we comprehensively simulated the models within this range. The model predictions captured the experimental trends, which had a single peak, indicating that our models successfully reproduced the tradeoff in the IPA production process with both strains.

### Controller performance against PMM

**Figure 8:**
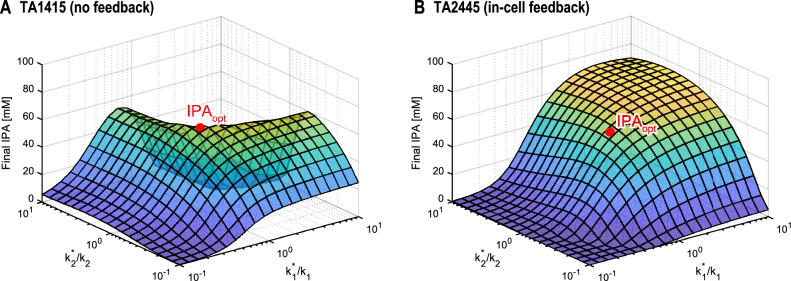
IPA yields in the presence of PMMs of various magnitudes were calculated in multiple simulation rounds. Each grid point on the surfaces represents the results of each round of simulations. On the x- and y- axes, ${k}_{1}^{*}$ and ${k}_{2}^{*}$ represent various values of the two cell growth parameters defined to calculate the process dynamics in each round of simulations. ${k}_{1}$ and ${k}_{2}$ represent the corresponding values defined in the controller model, which were, in contrast, fixed to the estimated values listed in Table 2 in all rounds of simulations. This means that IPTG input values were also constant in all rounds at the optimized values shown in Fig. 7 (IPTG adding time = 9.3 h for TA1415 and IPTG concentration = 0.044 mM for TA2445). Red dots represent the optimal IPA yields (i.e., IPA yields maximized when the cell growth rate parameters were defined equal between the controller model and in the actual process). (**A**) for TA1415. (**B**) for TA2445.

To evaluate the robustness of HISICC against PMM, we calculated the IPA yields of the two strains in multiple rounds of simulation (Fig. 8). In each round of simulations, represented as a grid point on the curved surfaces in Fig. 8, different magnitudes of the PMM were introduced, and the cell growth of both strains was assumed to be faster or slower than that predicted by the in silico feedforward controller. In contrast, in all simulation rounds, the controller models used for IPTG input optimization were identical to those trained using all experimental datasets (Fig. 5, Table 2). Therefore, the resulting IPTG input values in all simulation rounds were identical to those optimized in the model-based input optimization section (represented by dotted vertical lines in Fig. 7). To introduce the PMM into cell growth, for the two parameters (${k}_{1}$ and ${k}_{2}$ in Eqs. (6) and (7) for TA1415 and Eqs. (17) and (18) for TA2445, respectively) that determine the cell growth rate, we defined various combinations of values to compute the process dynamics. These values are denoted as ${k}_{1}^{*}$ and ${k}_{2}^{*}$ to distinguish them from the corresponding values in controller models ${k}_{1}$ and ${k}_{2}$. Note that ${k}_{1}^{*}$ and ${k}_{2}^{*}$ are parameters representing the intrinsic properties of the cells, which are difficult to artificially manipulate in real experiments. However, in this series of simulations, we set various values for these parameters to emulate possible situations in which the cell behavior deviates from that predicted by the in silico controller. In a round of simulations where $({k}_{1}^{*},{k}_{2}^{*})={(k}_{1},{k}_{2})$, no PMM was defined (represented by red dots in Fig. 8). This round represents an ideal situation where the in silico controller perfectly predicts cell behavior, and the IPA yield in this round is referred to as the optimal yield, ${\mathrm{IPA}}_{\mathrm{opt}}$. First, for both strains, when the growth rate was slower than prediction (namely ${k}_{1}^{*}/{k}_{1}<1\cap {k}_{2}^{*}/{k}_{2}<1$), IPA yields were lower than ${\mathrm{IPA}}_{\mathrm{opt}}$. This was apparently because cell density did not increase sufficiently during the fixed culture period. However, when the cell growth was faster than that predicted by the controller model, the two strains resulted in different IPA yields. In the case of TA1415 (which did not contain an in-cell feedback controller), the addition of IPTG was delayed relative to the truly optimal timing, resulting in a lower IPA yield than ${\mathrm{IPA}}_{\mathrm{opt}}$ (Fig. 8A). By contrast, in the case of TA2445, which contains an in-cell feedback controller, cells can autonomously adjust the timing of MTS activation earlier, resulting in suppression of the decrease in IPA yield (Fig. 8B). These results indicate that within HISICC, the in-cell feedback controller can support the in silico feedforward controller to prevent it from being disturbed by the PMM.

## Discussion

In this study, we proposed HISICC, a hybrid control system in which a high-level model-based controller provides a reference signal to a low-level in-cell feedback controller by means of the inducer concentration to suppress the performance deterioration caused by the PMM. We then performed a proof-of-concept of HISICC in the IPA production process with two *E. coli* strains that contain the MTS. Only one of these strains can be combined with the in silico feedforward controller to form a HISICC because it has an in-cell feedback controller that detects cell density (quorum sensing) to activate the MTS. We hypothesized that owing to the in-cell feedback controller, this HISICC can correct the timing of the MTS activation based on quorum sensing to prevent IPA yield decrease due to PMM of cell growth rate. To prove the hypothesis, first, mathematical models of the two strains were constructed to design an in silico feedforward controller. The constructed models are based on a previously reported two-compartment model. We used the experimental data from the IPA production culture to estimate the values of the parameters included in these models. Although the constructed models had simple structures, they captured the dynamics of cell growth and IPA production in response to various IPTG inputs. Both models showed excellent prediction performances for the experimental data in the hold-out validation. The validated models were then used to evaluate the robustness of HISICC against PMM. Finally, we compared the IPA yield between the two strains using simulations in which the model predictions and actual cell growth rates were assumed to be different. The results showed that, as we hypothesized, when cell growth is faster than expected by the in silico controller, the strain equipped with the in-cell feedback controller can prevent a decrease in IPA production. On the other hand, the strain without the in-cell controller cannot prevent a decrease in IPA production, which demonstrates the effectiveness of HISICC.

The cell density of the strain used in this proof-of-concept study was measured to autonomously activate the MTS. Since the cell density can be easily measured using a standard spectrophotometer instead of an in-cell feedback controller, it is easy to suppress the influence of the PMM by combining a low-level feedback controller using a spectrophotometer with a high-level model-based controller. However, as noted in the Introduction section, in many microbial processes, the optimization of process inputs involves intracellular concentrations of mRNA, proteins, metabolites, or products. In such cases, few biochemical analysis methods are applicable for feedback control of the process because of their long turnaround times. Bacteria-based processes require particularly short turnaround times due to rapid cell growth. We believe that the HISICC proposed in this study can be a solution to the PMM problem when the ongoing monitoring of the process state is challenging using conventional hard sensors or biochemical analysis methods.

Furthermore, we must note a few points regarding the three-compartment models that we constructed. First, the state variables included in these models are approximate and difficult to interpret as concentrations of specific substances. Williams discussed the same issue in his two-compartment model^34^. In particular, substrate S in our models does not correspond explicitly to the glucose concentration in the medium, but rather abstractly represents the total extracellular resources consumed for cell growth and enzyme synthesis, including nutrients such as sugars and nitrogen sources, accumulation of waste products, and pH shifts. These abstractions allow our models to capture the dynamics of cell growth and IPA production in response to various IPTG inputs, while maintaining very simple structures. Meanwhile, they limit the use of our model to off-line optimization of inducer addition, which was demonstrated in this study: since the state variables of our models cannot be directly associated with measured concentrations of substrates or metabolites in the medium, it is challenging to utilize the model for state estimation of an ongoing process or for model-based feedback control such as MPC.

Secondly, as mentioned in the model simulation and validation section, our model does not account for the slowdown in the production rate of IPA due to substrate depletion at the end stage of culture, as in the model reported by Dunlop et al*.*^27^. This approximation would have resulted in a higher yield of IPA than the optimal yield ${\mathrm{IPA}}_{\mathrm{opt}}$ in the simulation, which assumed that the actual cell growth was faster than that predicted by the in silico controller with TA2445 (Fig. 8B). Thus, the increase in the IPA yield owing to faster cell growth was negligible. However, we believe that this approximation does not affect our argument that HISICC prevents the reduction in IPA yield due to PMM.

In summary, we proposed the concept of HISICC that leverages the strengths of both in silico and in-cell controllers as a solution to the problem of PMM in bioprocesses and set and achieved the following two goals. First, we designed an example of HISICC in the IPA production process by combining a previously reported *E. coli* strain possessing an in-cell feedback circuit based on quorum sensing with an in silico feedforward controller based on a newly constructed mathematical model. The mathematical model exhibited high prediction performance for different process input values, indicating its feasibility for use in in silico feedforward controllers. Second, we demonstrated that HISICC can effectively compensate for the PMM through multiple rounds of simulations in which PMM of different magnitudes were intentionally introduced for cell growth. The proposed hybrid control strategy is expected to be applicable to various model-based optimizations and in-cell feedback circuits as a promising solution for PMM, which is a long-standing challenge in bioprocesses.

## Materials and methods

### Experimental data

The experimental data from the IPA production cultures used in this study to train and validate the models for the two engineered *E. coli* strains, TA1415 and TA2445, were obtained from two previously published studies^32,33^. Here, we provide a brief description of the IPA production culture experiments. For both strains, seed cultures were grown overnight in 3 mL of M9 minimal medium supplemented with 10 g/L glucose, 1 g/L casamino acids, and 10 ppm thiamine hydrochloride at 37 °C on a rotary shaker at 250 rpm. IPA production cultures were initiated with 1% (v/v) inoculation from the seed culture and grown in 20 mL of M9 minimal medium supplemented with 20 g/L glucose, 1 g/L casamino acids, and 10 ppm thiamin hydrochloride at 30 °C on a rotary shaker at 250 rpm. Cell density (OD600) and IPA concentration were measured routinely during culture. For TA1415, the culture duration was 69 h. In the middle of the culture, 0.1 mM IPTG (concentrated enough to activate the MTS) was added at five different timepoints (0, 6, 9, 12, and 15 h, Table S1). Three flasks were cultured for each addition of IPTG. For the TA2445 cells, the culture duration was 51.5 h. At the beginning of the culture period, different concentrations of IPTG (0.01, 0.03, 0.05, 0.1, or 1.0 mM, Table S1) were added to the medium to tune the in-cell feedback controller. Three flasks were cultured for each addition of IPTG.

### Parameter estimation

MATLAB/Simulink 2022a was used for model construction and simulation. In the modeling of TA2445, Curve Fitting Toolbox was used to approximate the promoter response to AHL and IPTG using the Hill equation, as described in the Mathematical modeling section. Simulink Design Optimization was used to estimate the other model parameters. The parameter values were chosen to minimize the sum of the squared errors between the model predictions and the measured data, as shown in Eqs. (23) and (24). Errors were normalized to the maximum values of measurements in the same culture. In these equations, $V$ represents the objective function for optimization. Vector ${\varvec{\theta}}$ and $\widehat{{\varvec{\theta}}}$ represent the model parameters and estimated values for them, respectively. $y$ and $\widehat{y}$ represent the measured and predicted process outputs, respectively. $\widetilde{u}$ represents the IPTG input (addition time for TA1415 and concentration for TA2445). The subscripts $i$, $j$, and $k$ represent the process output index ($i=1$ for cell density and $i=2$ for IPA concentration), culture flask index, and measurement time index, respectively.

23 $$\begin{array}{c}\widehat{{\varvec{\theta}}}=\mathrm{arg}\underset{{\varvec{\theta}}}{\mathrm{min}}\left[V\left({\varvec{\theta}}\right)\right]\end{array}$$

24 $$\begin{array}{c}V\left({\varvec{\theta}}\right)=\sum_{i=1}^{2}{\sum_{j}\sum_{k=1}^{N}\left(\frac{{y}_{i,j,k}-{\widehat{y}}_{i}\left({t}_{k};{\widetilde{u}}_{j}|{\varvec{\theta}}\right)}{\underset{{k}^{\mathrm{^{\prime}}}}{\mathrm{max}}\left[{y}_{i,j,{k}^{\mathrm{^{\prime}}}}\right]}\right)}^{2}\end{array}$$

The *lsqnonlin* command was used for optimization. The trust region method was selected as the optimization algorithm for the command. A scaling factor was specified for each parameter to prevent those with large absolute values from excessively influencing the overall parameter estimation.

### Model validation

We validated that the constructed models correctly predicted the cell density and IPA concentration in response to different IPTG input values using the hold-out method. For each round of validation, one IPTG input value was selected from the five experimental values, excluding the maximum and minimum values (Fig. 6). The experimental dataset from one of the three flasks to which the selected input condition was applied was defined as the validation dataset. The datasets from the remaining 12 flasks were collectively defined as the training datasets. For each round of validation, the coefficient of determination ${R}^{2}$ was calculated for the cell density or IPA concentration as follows:

$${{R}^{2}}_{i,j}=1-\frac{\sum_{k=1}^{N}{\left({y}_{i,j,k}-{\widehat{y}}_{i}({t}_{k};{\widetilde{u}}_{j}|{\widehat{{\varvec{\theta}}}}_{j})\right)}^{2}}{{\sum }_{k=1}^{N}{\left({y}_{i,j,k}-\frac{1}{N}{\sum }_{k\mathrm{^{\prime}}=1}^{N}{y}_{i,j,k\mathrm{^{\prime}}}\right)}^{2}}$$

$${\widehat{{\varvec{\theta}}}}_{j}=\mathrm{arg}\underset{{\varvec{\theta}}}{\mathrm{min}}\left[{V}_{j}({\varvec{\theta}})\right]$$

$${V}_{j}({\varvec{\theta}})={\sum }_{i=1}^{2}\sum_{j\mathrm{^{\prime}}\in {D}_{\mathrm{train},j}}{\sum }_{k=1}^{N}{\left(\frac{{y}_{i,j\mathrm{^{\prime}},k}-{\widehat{y}}_{i}({t}_{k};{\widetilde{u}}_{j\mathrm{^{\prime}}}|{\varvec{\theta}})}{\underset{k\mathrm{^{\prime}}}{\mathrm{max}}\left[{y}_{i,j\mathrm{^{\prime}},k\mathrm{^{\prime}}}\right]}\right)}^{2}$$

The subscripts $j=\mathrm{4,5},\dots ,\mathrm{12,19},\dots ,27$ represents the flasks selected for the validation dataset (Table S2). The subscript set ${D}_{\mathrm{train},j}$ represents the set of flasks selected for the training dataset.

### Model-based input optimization

Simulink Design Optimization was used to optimize the IPTG input. The optimal value ${\widetilde{u}}_{opt}$ was chosen to maximize the IPA concentration at the end of the culture, as shown in Eqs. (25) and (26). The culture duration ${t}_{N}$ in the simulation was defined as 69 h and 51.5 h for TA1415 and TA2445, respectively, as in the experiments.

25 $$\begin{array}{c}{\widetilde{u}}_{\mathrm{opt}}=\mathrm{arg}\underset{\widetilde{u}}{\mathrm{min}}\left[V\left(\widetilde{u}\right)\right]\end{array}$$

26 $$\begin{array}{c}V\left(\widetilde{u}\right)=-{\widehat{y}}_{2}\left({t}_{N};\widetilde{u}\right)\end{array}$$

### Supplementary Information

Supplementary Tables.

## Data Availability

The datasets and computer code used in this study are available at GitHub (https://github.com/kkunida/202304_Ohkubo_bioRxiv.git).

## References

[1] Villadsen J, Nielsen J, Lidén G (2011). Bioreaction Engineering Principles.

[2] Dochain, D. *Automatic Control of Bioprocesses*. (Wiley, 2008).

[3] Del Vecchio, D., Dy, A. J. & Qian, Y. Control theory meets synthetic biology. *J. R. Soc. Interface* **13**, (2016). 10.1098/rsif.2016.0380 PMC4971224 27440256

[4] Hsiao V, Swaminathan A, Murray RM (2018). Control theory for synthetic biology: recent advances in system characterization, control design, and controller implementation for synthetic biology. IEEE Control Syst. Mag..

[5] Khammash MH (2022). Cybergenetics: Theory and applications of genetic control systems. Proc. IEEE.

[6] Penloglou G, Vasileiadou A, Chatzidoukas C, Kiparissides C (2017). Model-based intensification of a fed-batch microbial process for the maximization of polyhydroxybutyrate (PHB) production rate. Bioprocess Biosyst. Eng..

[7] Majewski, R. A. & Domach, M. M. Simple constrained-optimization view of acetate overflow in *E. coli*. *Biotechnol. Bioeng.* **35**, 732–738 (1990). 10.1002/bit.260350711 18592570

[8] Novak M, Koller M, Braunegg M, Horvat P (2015). Mathematical modelling as a tool for optimized PHA production. Chem. Biochem. Eng. Q..

[9] Khanna S, Srivastava AK (2005). A simple structured mathematical model for biopolymer (PHB) production. Biotechnol. Prog..

[10] Horvat P (2013). Mathematical modelling and process optimization of a continuous 5-stage bioreactor cascade for production of poly[-(R)-3-hydroxybutyrate] by Cupriavidus necator. Bioprocess Biosyst. Eng..

[11] Milias-Argeitis A, Rullan M, Aoki SK, Buchmann P, Khammash M (2016). Automated optogenetic feedback control for precise and robust regulation of gene expression and cell growth. Nat. Commun..

[12] Hafidi, G., Tebbani, S., Dumur, D. & Vande Wouwer, A. Nonlinear model predictive control applied to *E. Coli* cultures. *IFAC Proc. 41*, 14570–14575 (2008).

[13] Santos LO, Dewasme L, Coutinho D, Wouwer AV (2012). Nonlinear model predictive control of fed-batch cultures of micro-organisms exhibiting overflow metabolism: Assessment and robustness. Comput. Chem. Eng..

[14] Tebbani, S., Dumur, D., Hafidi, G. & Vande Wouwer, A. Nonlinear predictive control of fed-batch cultures of *Escherichia coli*. *Chem. Eng. Technol.* **33**, 1112–1124 (2010).

[15] Uhlendorf J (2012). Long-term model predictive control of gene expression at the population and single-cell levels. Proc. Natl. Acad. Sci. U. S. A..

[16] Ashoori A, Moshiri B, Khaki-Sedigh A, Bakhtiari MR (2009). Optimal control of a nonlinear fed-batch fermentation process using model predictive approach. J. Process Control.

[17] Chang L, Liu X, Henson MA (2016). Nonlinear model predictive control of fed-batch fermentations using dynamic flux balance models. J. Process Control.

[18] Xiong Z, Zhang J (2005). Neural network model-based on-line re-optimisation control of fed-batch processes using a modified iterative dynamic programming algorithm. Chem. Eng. Process..

[19] Mahadevan, R. & Doyle, F. J., III. On-line optimization of recombinant product in a fed-batch bioreactor. *Biotechnol. Prog.* **19**, 639–646 (2003). 10.1021/bp025546z 12675609

[20] Hartline CJ, Schmitz AC, Han Y, Zhang F (2021). Dynamic control in metabolic engineering: Theories, tools, and applications. Metab. Eng..

[21] Izard J (2015). A synthetic growth switch based on controlled expression of RNA polymerase. Mol. Syst. Biol..

[22] Vignoni, A., Oyarzun, D. A., Pico, J. & Stan, G.-B. Control of protein concentrations in heterogeneous cell populations. in *2013 European Control Conference (ECC)* 3633–3639 (2013).

[23] You L, Cox RS, Weiss R, Arnold FH (2004). Programmed population control by cell–cell communication and regulated killing. Nature.

[24] Holtz WJ, Keasling JD (2010). Engineering static and dynamic control of synthetic pathways. Cell.

[25] Honjo H (2019). Synthetic microbial consortium with specific roles designated by genetic circuits for cooperative chemical production. Metab. Eng..

[26] Gutiérrez Mena, J., Kumar, S. & Khammash, M. Dynamic cybergenetic control of bacterial co-culture composition via optogenetic feedback. *Nat. Commun.* **13**, 1–16 (2022). 10.1038/s41467-022-32392-z PMC9381578 35973993

[27] Dunlop MJ, Keasling JD, Mukhopadhyay A (2010). A model for improving microbial biofuel production using a synthetic feedback loop. Syst. Synth. Biol..

[28] Harrison ME, Dunlop MJ (2012). Synthetic feedback loop model for increasing microbial biofuel production using a biosensor. Front. Microbiol..

[29] Gardner TS, Cantor CR, Collins JJ (2000). Construction of a genetic toggle switch in *Escherichia coli*. Nature.

[30] Hsiao, V., de los Santos, E. L. C., Whitaker, W. R., Dueber, J. E. & Murray, R. M. Design and implementation of a biomolecular concentration tracker. *ACS Synth. Biol.* **4**, 150–161 (2015). 10.1021/sb500024b PMC4384833 24847683

[31] Zhang F, Carothers JM, Keasling JD (2012). Design of a dynamic sensor-regulator system for production of chemicals and fuels derived from fatty acids. Nat. Biotechnol..

[32] Soma Y, Tsuruno K, Wada M, Yokota A, Hanai T (2014). Metabolic flux redirection from a central metabolic pathway toward a synthetic pathway using a metabolic toggle switch. Metab. Eng..

[33] Soma Y, Hanai T (2015). Self-induced metabolic state switching by a tunable cell density sensor for microbial isopropanol production. Metab. Eng..

[34] Williams FM (1967). A model of cell growth dynamics. J. Theor. Biol..

[35] Sekine R (2011). Tunable synthetic phenotypic diversification on Waddington’s landscape through autonomous signaling. Proc. Natl. Acad. Sci. U. S. A..

