# Supplementary material for: A hybrid in silico/in-cell controller for microbial bioprocesses with process-model mismatch
Source: Sci Rep. 2023 Sep 4;13:13608. doi: 10.1038/s41598-023-40469-y (PMC10477343; doi:10.1038/s41598-023-40469-y)
Supplement: Supplementary file 1 — Supplementary Tables. [file 41598_2023_40469_MOESM1_ESM.docx]

A hybrid *in silico*/in-cell controller
for microbial bioprocesses
with process-model mismatch

# Author information

Tomoki Ohkubo^1^, Yuki Soma^2^, Yuichi Sakumura^1,3^, Taizo Hanai^2^, Katsuyuki Kunida^1,4^*

1. Graduate School of Science and Technology, Nara Institute of Science and Technology, Ikoma, Nara, 8916-5, Japan
2. Laboratory for Synthetic Biology, Graduate School of Bioresource and Bioenvironmental Sciences, Kyushu University, W5-729, 744, Motooka, Nishi-ku, Fukuoka 819-0395, Japan
3. Data Science Center, Nara Institute of Science and Technology, Ikoma, Nara, 8916-5, Japan
4. School of Medicine, Fujita Health University, Toyoake, Aichi, 470-1192, Japan

*Correspondence: [katsuyuki.kunida@fujita-hu.ac.jp](mailto:katsuyuki.kunida@fujita-hu.ac.jp)

# Supplementary information

Table S1. The composition of the experimental datasets.

| Experimental conditions | | Culture flask # |
| --- | --- | --- |
| Strain | IPTG input value |  |
| **TA1415** | **0 h** | 1, 2, 3 |
|  | **6 h** | 4, 5, 6 |
|  | **9 h** | 7, 8, 9 |
|  | **12 h** | 10, 11, 12 |
|  | **15 h** | 13, 14, 15 |
| **TA2445** | **0.01 mM** | 16, 17, 18 |
|  | **0.03 mM** | 19, 20, 21 |
|  | **0.05 mM** | 22, 23, 24 |
|  | **0.1 mM** | 25, 26, 27 |
|  | **1.0 mM** | 28, 29, 30 |

Table S2. Partitioning of datasets for hold-out validation.

|  |  |  | **Validation dataset** | | **Training dataset** | |
| --- | --- | --- | --- | --- | --- | --- |
| Strain | Measurement | Round | IPTG input | Culture flask# | IPTG input | Culture flask # |
| **TA1415** | **OD600** | 1 | **6 h** | 4 | **0, 9, 12, 15 h** | 1,2,3,7,8,9,10,11,12,13,14,15 |
|  |  | 2 |  | 5 |  |  |
|  |  | 3 |  | 6 |  |  |
|  |  | 4 | **9 h** | 7 | **0, 6, 12, 15 h** | 1,2,3,4,5,6,10,11,12,13,14,15 |
|  |  | 5 |  | 8 |  |  |
|  |  | 6 |  | 9 |  |  |
|  |  | 7 | **12 h** | 10 | **0, 6, 9, 15 h** | 1,2,3,4,5,6,7,8,9,13,14,15 |
|  |  | 8 |  | 11 |  |  |
|  |  | 9 |  | 12 |  |  |
|  | **IPA** | 10 | **6 h** | 4 | **0, 9, 12, 15 h** | 1,2,3,7,8,9,10,11,12,13,14,15 |
|  |  | 11 |  | 5 |  |  |
|  |  | 12 |  | 6 |  |  |
|  |  | 13 | **9 h** | 7 | **0, 6, 12, 15 h** | 1,2,3,4,5,6,10,11,12,13,14,15 |
|  |  | 14 |  | 8 |  |  |
|  |  | 15 |  | 9 |  |  |
|  |  | 16 | **12 h** | 10 | **0, 6, 9, 15 h** | 1,2,3,4,5,6,7,8,9,13,14,15 |
|  |  | 17 |  | 11 |  |  |
|  |  | 18 |  | 12 |  |  |
| **TA2445** | **OD600** | 1 | **0.03 mM** | 19 | **0.01, 0.05, 0.1, 1.0 mM** | 16,17,18,22,23,24,25,26,27,28,29,30 |
|  |  | 2 |  | 20 |  |  |
|  |  | 3 |  | 21 |  |  |
|  |  | 4 | **0.05 mM** | 22 | **0.01, 0.03, 0.1, 1.0 mM** | 16,17,18,19,20,21,25,26,27,28,29,30 |
|  |  | 5 |  | 23 |  |  |
|  |  | 6 |  | 24 |  |  |
|  |  | 7 | **0.1 mM** | 25 | **0.01, 0.03, 0.05, 1.0 mM** | 16,17,18,19,20,21,22,23,24,28,29,30 |
|  |  | 8 |  | 26 |  |  |
|  |  | 9 |  | 27 |  |  |
|  | **IPA** | 10 | **0.03 mM** | 19 | **0.01, 0.05, 0.1, 1.0 mM** | 16,17,18,22,23,24,25,26,27,28,29,30 |
|  |  | 11 |  | 20 |  |  |
|  |  | 12 |  | 21 |  |  |
|  |  | 13 | **0.05 mM** | 22 | **0.01, 0.03, 0.1, 1.0 mM** | 16,17,18,19,20,21,25,26,27,28,29,30 |
|  |  | 14 |  | 23 |  |  |
|  |  | 15 |  | 24 |  |  |
|  |  | 16 | **0.1 mM** | 25 | **0.01, 0.03, 0.05, 1.0 mM** | 16,17,18,19,20,21,22,23,24,28,29,30 |
|  |  | 17 |  | 26 |  |  |
|  |  | 18 |  | 27 |  |  |
